# Disposition of Aerosols of Isothiazolinone-Biocides: BIT, MIT and OIT

**DOI:** 10.3390/toxics10120770

**Published:** 2022-12-10

**Authors:** Seungmi Lee, Heui-Jin Park, Eunice B. Lee, Do Hyeon Lee, Dalwoong Choi, Kyung-Min Lim

**Affiliations:** 1 College of Pharmacy, Ewha Womans University, Seoul 03760, Republic of Korea; 2 Department of Chemistry and Biochemistry, University of California, Los Angeles (UCLA), Los Angeles, CA 90095, USA; 3 Transdisciplinary Major in Learning Health Systems, Department of Health and Safety Convergence Science, Korea University, Seoul 02481, Republic of Korea

**Keywords:** biocide, isothiazolinone, MIT, OIT, BIT, aerosols

## Abstract

Biocides are widely used in everyday life, and accordingly, human exposure to them is inevitable. Especially, the inhalational exposure of humans to biocides and resultant respiratory toxicity are gaining public interest due to the recent catastrophe associated with humidifier disinfectants. Aerosolized chemicals are subject to gravitational deposition and chemical degradation. Therefore, the characterization of the disposition of aerosols is essential to estimate the inhalational exposure to biocides. Here, we compared the disposition of aerosols of one of the commonly used biocide classes, isothiazolinone-based biocides, BIT, MIT, and OIT. An acrylic chamber (40 cm × 40 cm × 50 cm) was created to simulate the indoor environment, and a vacuum pump was used to create airflow (1 LPM). Biocides were sprayed from a vertical nebulizer placed on the ceiling of the chamber, and the distribution of particle sizes and volume was measured using the Optical Particle Sizer (OPS) 3330 device. During and after the aerosol spraying, airborne biocides and those deposited on the surface of the chamber were sampled to measure the deposition using LC-MS/MS. As a result, the broad particle size distribution was observed ranging from 0.3 to 8 μm during the nebulization. The inhalable particle faction (>2 μm) of the isothiazolinones was 32–67.9% in number but 1.2 to 6.4% in volume. Most of the aerosolized biocides were deposited on the chamber’s surface while only a minimal portion was airborne (<1%) after the nebulization. More importantly, significant amounts of MIT and OIT were degraded during aerosolization, resulting in poor total recovery compared to BIT (31%, 71% vs. 97% BIT). This result suggests that some isothiazolinones may become unstable during nebulization, affecting their disposition and human exposure significantly.

## 1. Introduction

In our everyday life, biocides are used in a variety of consumer products like cleaning preparations for household use (e.g., wet tissues, hand sanitizers, detergents, and humidifiers), disinfectants for surface decontamination [1], and as preservatives of various consumer products [2]. However, biocides carry potential risks to human health, as can be exemplified by a recent catastrophe due to humidifier disinfectants in South Korea [3]. Hundreds of consumers have suffered from respiratory diseases such as interstitial pneumonia and lung fibrosis, which are attributable to the inhalation of aerosolized humidifier disinfectants [4]. This accident was caused by the inadvertent use of biocides, of which potential impacts on respiratory systems via inhalational exposure had not been properly evaluated.

Isothiazolin-3-ones are one of the most widely used biocide classes to prevent fungal growth in various consumer chemical products, such as emulsion paints, wood varnishes, adhesives, and natural and artificial leather [5]. Isothiazolinone-based compounds manifest biocidal activities through the interaction of nitrogen–sulfur (N–S) bonds of the isothiazolinone ring with thiol groups (R–SH) of the cell membrane proteins of the micro-organisms [6,7]. The oxidized thiol group leads to the dysfunction of the pivotal proteins and free radical generation which can result in microbial cell death. Furthermore, isothiazolinone biocides inhibit microbial growth and metabolism by interfering with the mitochondrial production of adenosine triphosphate (ATP) [6].

The most well-known isothiazolinone biocides are 5-chloro-2-methyl-4-isothiazolin-3-one (CMIT) and 2-methyl-4-isothiazolin-3-one (MIT). They were developed in the 1960s by Dow Enterprises (Rohm and Hass, Little Rock, AR, USA, at the time). CMIT/MIT (3:1) and MIT have been widely used in cosmetics as active ingredients of preservatives. With the expanded use of CMIT/MIT over the years, concerns related to their inherent skin and respiratory sensitization have been reported [8,9]. Consequently, CMIT/MIT was banned from several countries including Korea [10], but MIT is still in use. Briefly, 1,2-benzisothiazolin-3-one (BIT), which was introduced in 1960 [8], is applied to the preservation of industrial and personal care products [11]. BIT is widely detected in household products such as laundry detergent, fabric softener, and shampoo. Under the EU regulation, BIT is prohibited for use in cosmetic products [12] but it is still in use in many countries. BIT is also used in industrial products, the most frequent products are paints, cutting oils, and leather products [13]. There are several cases of allergic contact dermatitis caused by BIT. Cases of occupational allergic contact dermatitis due to BIT have been reported in workers using paints and healthcare workers using medical PVC (polyvinyl chloride) gloves [8]. Additionally, the inhalation of BIT has been ascribed to occupational asthma and rhinitis in chemical industry workers [14]. Furthermore, 2-n-octyl-4-isothiazolin-3-one (OIT) is another commonly used isothiazolinone biocide. OIT is highly permeable to the skin following dermal exposure [9]. Contact dermatitis induced by OIT following occupational or non-occupational exposure has been reported in humans [15,16,17], suggesting potential adverse effects on the lung when inhaled.

Interestingly, isothiazolinones including CMIT/MIT are susceptible to photolysis and oxidation [18], and we also observed that isothiazolinones became less toxic to reconstructed airway tissues when they were exposed to aerosols than to neat solution [19]. These suggest that during aerosolization, the concentration of biocides in the atmosphere may decrease more than theoretically estimated, reflecting that it is necessary to investigate the disposition of isothiazolinone biocides after aerosolization to accurately estimate human exposure to biocide aerosols. Here, we aimed to compare the disposition of BIT, MIT, and OIT in aerosolized forms using a medical-grade nebulizer (ultrasonic-based) in an in-house built chamber to simulate the environment of daily humidifier use through LC-MS/MS analysis. We also determined the particle characteristics of biocide aerosols using an optical particle sizer spectrometer (OPSS) which measures aerosol particle size distribution to estimate the inhalational exposure to biocides.

## 2. Materials and Methods

### 2.1. Biocides and Reagents

MIT, BIT, and OIT were purchased from Sigma-Aldrich (St. Louis, MO, USA). 

### 2.2. Chamber and Instruments

**Figure 1:**
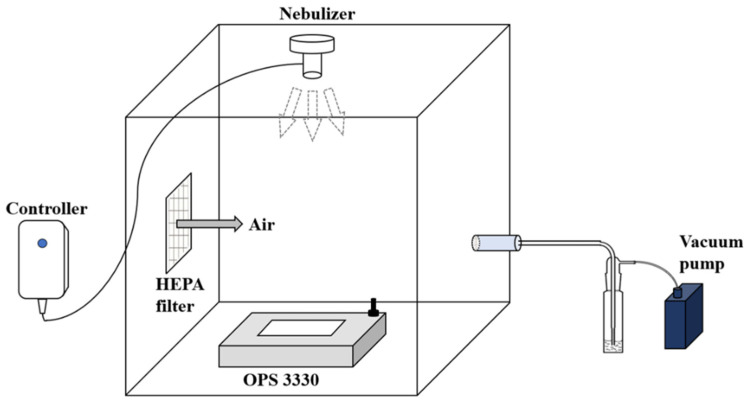
Aerosol exposure system of humidified biocides to the OPSS through a nebulizer.

We purchased the Aerogen^®^ Pro nebulizer from Aerogen (AG-AP6000-IN, Galway, Ireland) to generate aerosolized biocides. According to the product insert, the average flow rate of the nebulizer is 0.4 mL/min and the particle size range is 1–6.2 μm. The amount of aerosol varies according to the nebulization rate and the kind of materials. An optical particle sizer (OPS, model 3330, TSI Inc., Shoreview, MN, USA) was used to measure the particle concentration and distribution. The chamber used was an acryl box of size 40 × 40 × 50 cm (Figure 1). A hole was formed in the upper side of the chamber that fitted the nebulizer, another for a HEPA filter on one side of the chamber, and another on the opposite side of the HEPA filter that was connected to a vacuum pump.

### 2.3. Sampling of Aerosolized Biocides

**Figure 2:**
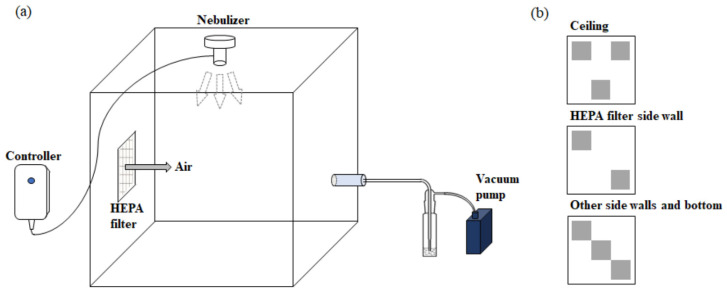
(**a**) Chamber setting for sampling aerosolized biocides with aluminum foil. (**b**) Setup of the aluminum foils (10 × 10 cm) on each side of the chamber.

The characteristics of the aerosols of BIT, MIT, and OIT were measured by OPS model 3330 located inside the chamber (40 × 40 × 50 cm^3^, ~80 L). The nebulizer was used to generate biocide, and an artificial flow of air was generated through a HEPA filter using a vacuum pump (1 L/min, LPM) (Figure 2). Briefly, 0.25 mL of biocide solution (1% DMSO spiked in DDW) was nebulized for 1 min. The particle number, size, and volume of the humidified biocides were measured through the OPSS for 3 min. 

### 2.4. Sampling of the Precipitated and Airborne Fraction of Aerosolized Biocides

The precipitation of the aerosols of BIT, MIT, and OIT was monitored by sampling the deposited aerosol particles on the upper, sides, and bottom of the chamber using sampling aluminum foil sheets (10 × 10 cm). We checked the stability of the BIT, MIT, and OIT on the aluminum foil sheets. Settled isothiazolinone particle samples were extracted and quantitated as described below. The inner surface area of the chamber was considered, and the amounts of isothiazolinone collected on the aluminum foil sheets were extrapolated to obtain the total isothiazolinone precipitated or adsorbed on the chamber surface [e.g., to obtain the isothiazolinone adsorbed to the ceiling, 1600 cm^2^ (the area of the ceiling)/three aluminum foils (300 cm^2^) was multiplied to the isothiazolinone sampled on 3 aluminum foils]. Airborne particles were sampled through a polytetrafluoroethylene (PTFE) filter (0.22 μm) and an impinger filled with 20 mL methanol (Figure 2). Briefly, 0.25 mL of biocide solution (1% DMSO spiked in DDW) was nebulized for 1 min. We waited for the settlement of the aerosols for further 2 min and collected the samples. The recovery of the aerosols was calculated.

### 2.5. Sample Extraction and Calibration Sample Preparation

The sampled aluminum foils were soaked in 10 mL methanol and extracted under gentle shaking in a Petri dish for 1 h. The samples collected in the PTFE filter were flushed with 10 mL methanol with a syringe. After extraction, samples were aliquoted into microtubes and subjected to centrifugation at 13,000× *g* for 10 min (Sorvall RC 6 Plus centrifuge, Thermo Fisher Scientific Inc., Waltham, MA, USA equipped at Ewha Drug Development Research Core Center). The supernatant was filtered using a 0.22 μm polytetrafluoroethylene (PTFE) filter (ADVANTEC, Dublin, CA, USA).

Calibration standards were prepared at 0.977, 1.953, 3.906, 7.812, 15.625, 31.25, 62.5, 125, 250, and 500 ng/mL by adding a standard solution to a blank matrix. All calibration standards were extracted in the same way as the other analytes before analysis. The samples were properly diluted such that the concentration fell within the calibration ranges.

### 2.6. Liquid Chromatography-Mass Spectrometry (LC-MS/MS)

A Waters ACQUITY ultra-high-performance liquid chromatography system (Waters, Milford, MA, USA) equipped with a Quantiva triple quadrupole mass spectrometer (Thermo Scientific, San Jose, CA, USA) was used to analyze the isothiazolinone biocides. An Agilent ZORBAX Eclipse Plus C18 column (2.1 × 50 mm, 1.8 µm) was installed in the instrument. The analyses were carried out using 0.1% formic acid in ultra-pure water as the “A” mobile phase and methanol in the “B” mobile phase at a flow rate of 0.3 mL/min. The column temperature was set to 40 °C, and the injection volume was 1 μL. The mobile phase gradient was started at 30% B, and a linear gradient was applied to increase eluent B to 100% over 10 min, where it was held for 3 min. 

An electrospray ion source (ESI) was used to ionize the isothiazolinone biocides. The ion source temperature was set at 350 °C, cone gas flow, 20 psi, heated probe temperature, 250 °C; probe gas flow, 45 psi, and nebulizer gas flow, 55 psi. The spray voltage was set to 4500 V. Xcalibur 4.1.50 (Thermo Scientific, San Jose, CA, USA) software was used for data processing. 

**Figure 3:**
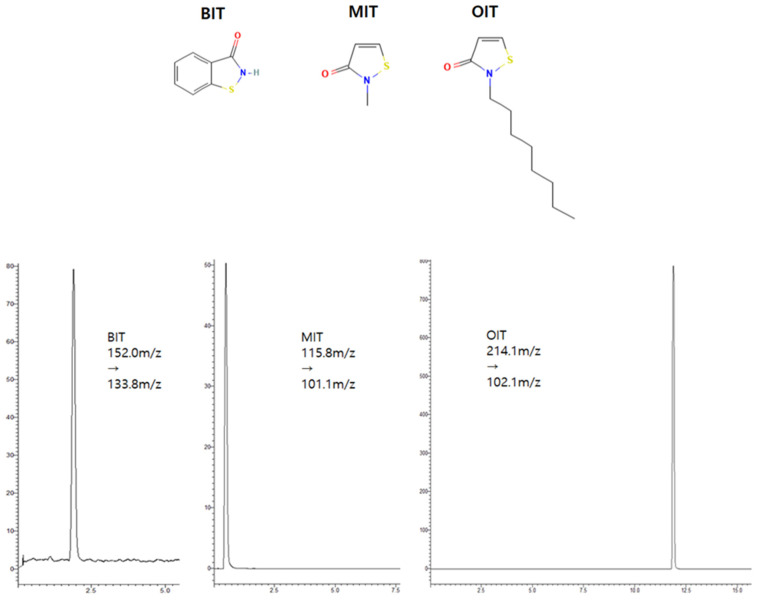
Structure and representative LC-MS/MS chromatograms of BIT, MIT, and OIT at 500 ng/mL.

The BIT, MIT, and OIT were monitored using MRM (multiple reaction monitoring) with mass transition set for BIT as 152.0 *m*/*z* → 133.8 *m*/*z* (22.0 V), MIT: 115.8 *m*/*z* → 101.1 *m*/*z* (22.0 V) and OIT: 214.1 *m*/*z* → 102.1 *m*/*z* (11.0 V). Representative chromatograms are shown in Figure 3.

### 2.7. Validation of the Analytical Method

The validation of the analytical method was confirmed through linearity, recovery rate, and precision using a calibration curve and QC samples. All calibration curves were generated by a regression method of the peak area ratio among different concentrations of calibration standards. The coefficient of determination (R^2^) for the calibration curves ranged from 0.993 to 0.999. The method detection limit (MDL) was analyzed by the pre-treating standard, and the value of the lowest concentration was selected where the signal-to-noise ratio (S/N) of the detected analyte was 3. The measured MDL values for the BIT, MIT, and OIT were 31.25 ng/mL, 1.953 ng/mL, and 1.953 ng/mL, respectively.

For the recovery rate and precision between days, the sample (n = 3) spikes in each blank matrix at concentrations of 62.5, 125, and 250 ng/mL were analyzed and performed on three consecutive days. The precision was expressed as a percentage of relative standard deviation (% RSD).

### 2.8. Data Analysis

Data are expressed as the mean ± SD.

## 3. Results 

### 3.1. Particle Characterization of Aerosols of BIT, MIT and OIT 

**Figure 4:**
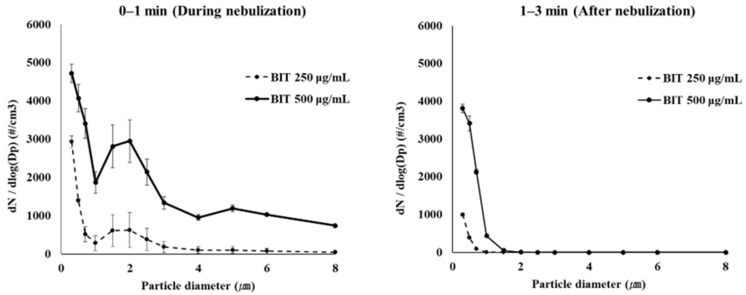
Particle size distribution of BIT aerosols (250 and 500 μg/mL) in number measured in an in-house-built exposure chamber.

The size distribution of aerosol particles is an important determinant of their deposition in the human lung after inhalation. In humans, most particles with sizes larger than 10 μm are filtered in the nose or oral pharynx and cannot reach the tissues beyond the larynx [20]. To examine the particle size distribution of biocide aerosols, we tried to measure the particle size distribution of the aerosols of BIT, MIT, and OIT in an in-house-built exposure chamber (40 × 40 × 50 cm^3^, ~80 L, Figure 4). Firstly, to examine the effect of biocide concentration on the particle size distribution, BIT was dissolved in 0.25 mL DW (1% DMSO) at 250, and 500 μg/mL and aerosolized for 1 min using the nebulizer, and the particle size distribution was measured during the nebulization and 1–3 min after the cessation of the nebulization. As the results show in Figure 4, the number of particles was observed to be larger at the higher concentration, suggesting that aerosolized particles may be mostly composed of BIT rather than water.

**Figure 5:**
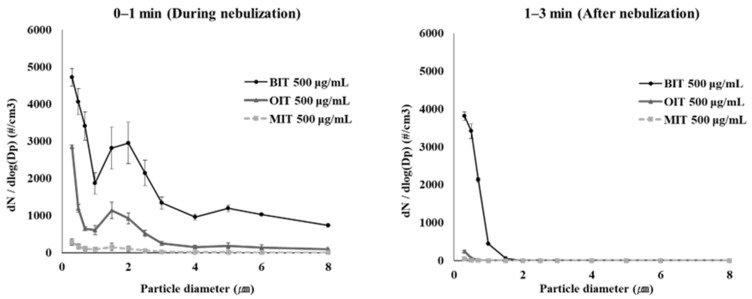
Particle distribution of BIT, OIT and MIT aerosols (at 500 μg/mL) in number were measured in an in-house-built exposure chamber.

Next, to compare the particle characteristics depending on the type of isothiazolinone, BIT, MIT, and OIT were dissolved in 0.25 mL DW (1% DMSO) at 500 μg/mL and aerosolized for 1 min using the nebulizer, and the particle size distribution was measured during the nebulization and 1–3 min after the cessation of the nebulization. As shown in Figure 5, most of the particles in the chamber air fell below 10 μm, and the particles below 1 μm constituted the majority of the aerosol particles in the air. However, the number of particles of sizes > 1 μm rapidly decreased after the nebulization while particles with smaller sizes < 2 μm remained relatively longer in the air after nebulization.

**Figure 6:**
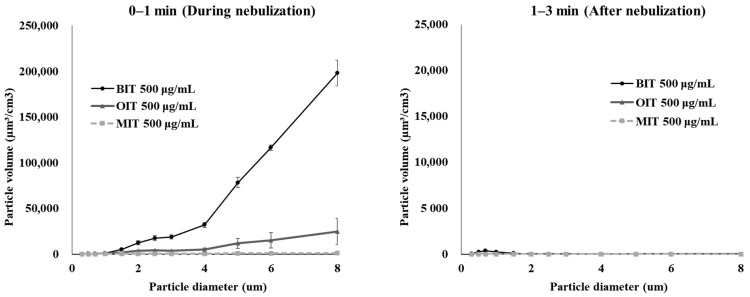
Particle distribution of BIT, MIT, and OIT aerosols in volume measured in an in-house-built exposure chamber.

This phenomenon could be observed more evidently when the sizes and numbers of the aerosol particles were calculated into the particle distribution in volume as shown in Figure 6.

The particles of 1–3 μm size contributed greatest to the total particle volume of biocide aerosols during nebulization, but after the cession of nebulization, the volume of particles remaining in the air reduced significantly, most probably due to the precipitation. The size-dependent particle distribution in terms of particle number and volume is shown in Table 1.

Based on the volume distribution of aerosol particles according to size, the inhalable fraction of the biocide aerosols that can reach beyond the larynx (particle size ≤ 2 μm [21]) could be calculated to be 32%, 57.2%, and 67.9% in particle number or 1.2%, 3.0%, and 6.4% in particle volume for aerosolized BIT, MIT and OIT, respectively, suggesting that only a minor portion of the aerosolized isothiazolinones were available for inhalation. 

### 3.2. Deposition of Aerosols of BIT, MIT, and OIT 

Humans can be exposed to aerosolized biocides through direct dermal contact besides inhalation during the use of spray or trigger-type biocidal products. To estimate the fraction of the aerosolized biocides available for dermal absorption or inhalation, the disposition of the aerosolized isothiazolinones was investigated. To accurately estimate the exposed dose, quantitative analysis was performed for the sampled aerosols using LC-MS/MS analysis. The precipitated aerosols of BIT, MIT, and OIT on the surface were monitored by sampling the deposited aerosol particles on the upper, sides, and bottom of the chamber using aluminum foil sheets (10 × 10 cm). Airborne particles were sampled through a polytetrafluoroethylene (PTFE) filter (0.22 μm) and an impinger linked with a vacuum pump with a flow rate of 1 L/min.

**Figure 7:**
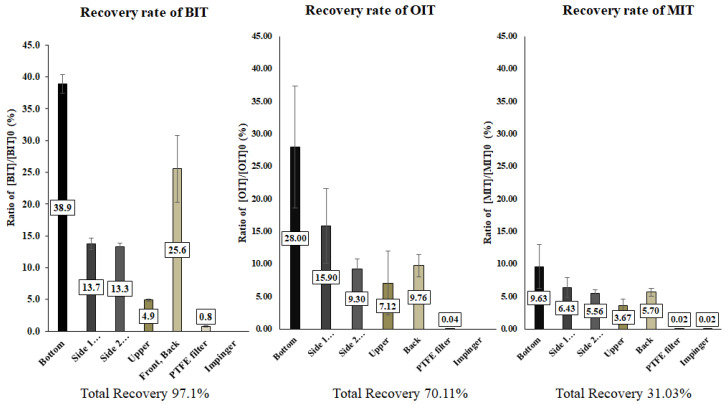
Disposition of biocide aerosols in the exposure chamber. The aerosolized isothiazolinones deposited on the chamber surface and escaped through the air flow (PTFE filter and an impinger) were collected and analyzed.

As shown in Figure 7, the disposition of isothiazolinones differed across each isothiazolinone. In the case of BIT, most of the aerosolized BIT (97.1%) was recovered predominantly from the surface precipitation (96.3%), suggesting that most of the aerosolized biocides were amenable to dermal absorption, which matches well with the results of the particle size distribution. For OIT and MIT, however, the total recovery was much lower than that of BIT. While the relative recovery from surfaces, the PTFE filter, and the impinger was similar, the total recovery of OIT and MIT was much lower (70.11% and 31.03%) than BIT, reflecting that a significant portion of OIT and MIT was degraded during aerosolization.

## 4. Discussion

Here, we compared the disposition of aerosols of a commonly used biocide class, isothiazolinone-based biocides, BIT, MIT, and OIT, using an in-house-built bench-top chamber and a medical nebulizer. A broad particle size distribution was observed ranging from 0.3 to 8 μm during the nebulization. Inhalable particle faction (>2 μm) of isothiazolinones was 32–67.9% in number but 1.2 to 6.4% in volume, indicating that only a small portion of the generated aerosols may be amenable to inhalation exposure in humans. Furthermore, most of the aerosolized biocides were deposited on the surface of the chamber, while only a minute portion in volume was airborne (<1%) after the nebulization. More notably, significant amounts of MIT and OIT were found to be degraded during aerosolization, resulting in poor total recovery compared to BIT (31% and 71% vs. 97% BIT). This poor recovery was well in line with the smaller particle numbers and volumes of MIT and OIT aerosols in the air, even though the same amount was nebulized into the chamber, suggesting that some isothiazolinones may become unstable during nebulization, affecting their disposition and exposure significantly.

Most of the aerosolized isothiazolinones were deposited on the surface of the chamber, limiting the direct inhalation of isothiazolinone aerosols. However, although the deposited isothiazolinone biocides could not be inhaled directly, they could be exposed through the inhalation of biocides carried over by evaporating water vapors [22,23]. Recent studies have revealed that indoor air quality can be worsened and hazardous to human health by mixtures of volatile chemicals and non-volatile chemicals carried airborne by water vapors [24]. Surprisingly, the ionic repulsion at the air–water interface enables the solvation of hydrophobic substances [25], which are often toxic to human health. Regrettably, we only monitored the aerosols for a short time, but an evaluation of air concentrations for an extended time will be necessary to check the possible air contamination from the deposited isothiazolinones. Furthermore, there is a chance of dermal exposure via skin contact with dust or water droplets [26]. We recently showed that BIT is fairly well permeable to the skin [27]. We confirmed that MIT and OIT are also skin-permeable (unpublished data), supporting the possible contribution of the dermal route for exposure to isothiazolinone aerosols. 

We demonstrated that the aerosolization of 500 μg/mL BIT produces more aerosol particles in the air than 250 μg/mL BIT, suggesting that the concentration of biocides as the solutes of supplying solutions is a key factor determining the air concentration of aerosol particles. Actually, it is foreseeable since most of the water in the aqueous aerosol particles will evaporate upon aerosol production while the solutes remain as airborne particles. Furthermore, the presence of solutes in the aerosol particles deters water evaporation by affecting the diffusion of water [28]. Therefore, using highly concentrated biocide aerosols may result in the production of a larger number of aerosol particles in the air, which may increase the inhalational exposure of humans to biocides.

The chemical reactivity and resultant instability of isothiazolinones have been reported previously [18,29]. Isothiazolinones can undergo isomerization, oxidation, hydroxylation, hydrolysis, and elimination processes, which can reduce their biocidal activity as well as their toxicity. Further supporting the inherent instability of isothiazolinones, toxicity tests of BIT on zebrafish embryos revealed that the toxicity of BIT decreases during oxonization, which was attributable to the oxidation of the reductive sulfur in BIT [30]. Although chemical reactivity appears common to isothiazolinones, the extent of the reactivity seems to vary depending on the individual chemical. Bollmann et al. examined the degradation of isothiazolinones in soil, which demonstrated that MIT is rapidly degraded with the shortest half-life of 0.28 days, followed by BIT (0.52 days) and OIT (9.3 days) [31]. Actually, isothiazolinones are far more susceptible to soil degradation compared to other classes of biocides, and the authors suggest that there is little concern about their bioaccumulation in the environment. 

The degradation of isothiazolinones depends on the presence of nucleophiles to react with the sulfur atom in the isothiazole ring, such as metals, amines, thiols, and sulfides. Upon the degradation, the five-membered isothiazole ring opens and isothiazolinones lose their biocidal activity due to the loss of electron-deficient sulfur atoms to react with endogenous nucleophiles. Aerosols are sprayed into the oxygen-rich environment, and accordingly, there is a high chance of oxidation on the surface of aerosols [32,33]. Furthermore, the pH at the core of aqueous aerosols increases up to pH 12, which enables or promotes the catalytic reaction favoring basic conditions [34]. Interestingly, isothiazolinones become vulnerable to hydrolysis at higher pHs and temperatures [35]. In the alkaline status, OH- attacks the electron-deficient sulfur atom and leads to the ring opening [36]. At pH 9, the degradation rate of CMIT increases by over 2000-fold compared to neutral pH [37], which may explain the accelerated degradation of MIT and OIT in the aerosol states, at least in part. 

Collectively, we demonstrated that most of the isothiazolinone aerosols deposited on the surface due to precipitation limiting the direct inhalational exposure. Additionally, we showed that isothiazolinones may become unstable during aerosolization depending on the structure, which may affect their disposition and human exposure significantly. Considering that the standard inhalation toxicity testing in rodents requires enormous resources and efforts [38], exposure assessment using a benchtop chamber may provide an important tool to estimate the particle size distribution and disposition of aerosolized chemicals and resultant human exposure. Although further studies are necessary, we believe that our study may shed a light on the understanding of the inhalational exposure and toxicity of isothiazolinone biocides. 

## Figures and Tables

**Table 1:** The fraction of the particle number and the volume of biocide aerosols during the nebulization.

Particle Size (μm)	Number of Aerosol Particles (% of total)			Volume of Aerosol Particles (% of total)		
	BIT	MIT	OIT	BIT	MIT	OIT
<0.3	-	-	-	0.0	0.0	0.0
0.3–1.0	19.7	25.7	30.7	0.1	0.1	0.3
1.0–2.0	21.3	31.5	37.2	1.1	2.9	6.1
2.0–4.0	26.8	24.8	22.8	8.2	11.5	18.0
>4.0	32.2	18.0	9.3	90.6	85.5	75.5

## Data Availability

The data presented in this study are available on request from the corresponding author.
